# Case of Congenital Artificial Joint in Ossa Femores

**Published:** 1837

**Authors:** William Judkins

**Affiliations:** Cin.


Art. III.—A Case of Congenital Artificial Joint in the Os Femoris of each Limb. Communicated by William Judkins, M. D.

Several years ago, I attended E. R. in her second labor.—The presentation was natural, and the delivery effected in due time, without accident or the interposition of art. Immediately after the birth of the child, a female, I observed that it threw its feet back against its ears, and kept them so, without extending or moving them in any other direction.

On examination I found that each thigh bone had a transverse solution of continuity, a little below its middle, and that the knee joints were in a state of immobility or anchylosis. The inferior extremity of the upper half of each bone, was rounded, and felt much like the olecranon process, when the arm is flexed. When I pulled by the feet, the limbs were easily extended; but immediately reverted to the trunk of the body.

I determined to begin the treatment of this case immediately, and after the child was washed, proceeded in the following manner:—Extending the limbs, I covered both thighs with adhesive plaster, and then applied splints and rollers. The bones were thus without difficulty kept in coaptation. I then broke up the adhesion of the cartilages, in the knee joints, and directed the nurse to move them several times a day.

By the end of the month, I renewed the dressings, and found the bones united; and by this time the child began to use its knee joints very well. It learned to walk at the usual age; and now, when 10 years old, has the perfect use of both limbs.

*Cin., 2d mo.* 1837.

